# How does ROP develop?

**Published:** 2017

**Authors:** Andrea Molinari, Dan Weaver, Subhadra Jalali

**Affiliations:** Pediatric Ophthalmologist: Hospital Metropolitano, Av. Mariana de Jesus Oe-8, Quito, Ecuador.; Pediatric Ophthalmologist: Billings Clinic, Billings, Montana, USA.; Deputy Director: Newborn Eye Health Alliance (NEHA) and Director, Quality: LV Prasad Eye Institute, Hyderabad, India.

**Figure:**
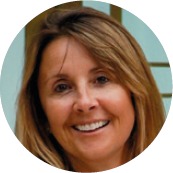
Andrea Molinari

**Figure:**
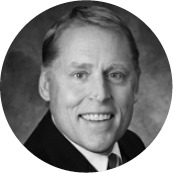
Dan Weaver

**Figure:**
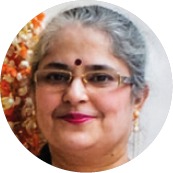
Subhadra Jalali

**Retinopathy of prematurity can develop when babies are born before their retinal blood vessels are fully formed.**

**Figure 1:**
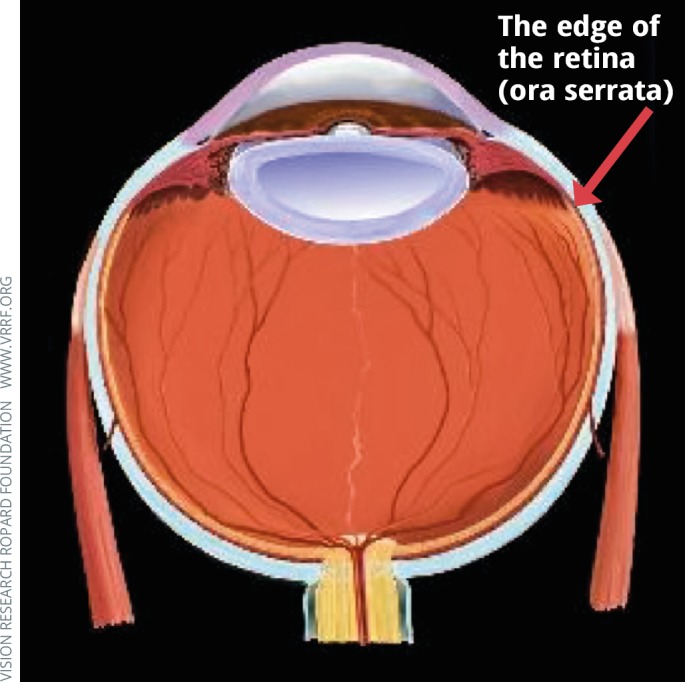
In the full-term eye, the retinal blood vessels are fully developed

In babies who are born at full term (between 37 and 42 weeks of gestation), the retinal blood vessels are fully developed and reach the edge of the retina: the ora serrata (Figure 1).

**Figure 2:**
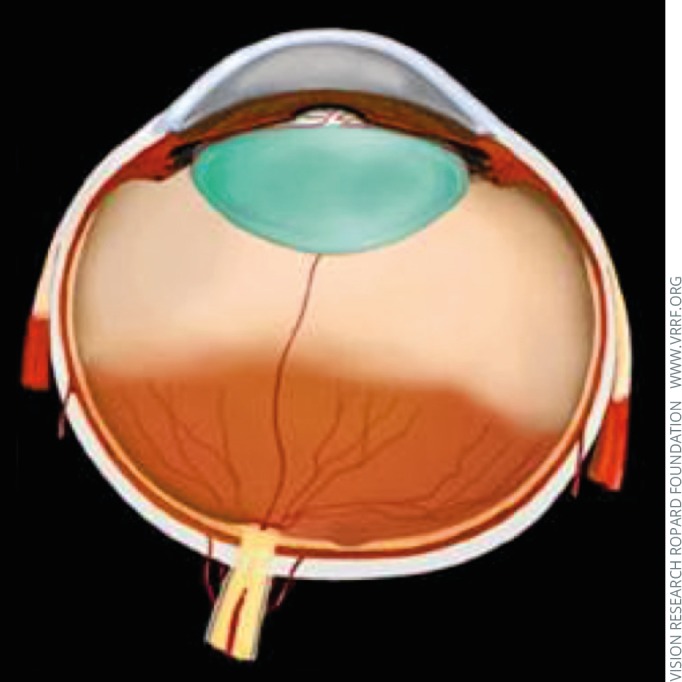
In the preterm eye, the retinal blood vessels are not fully developed

**Figure 3:**
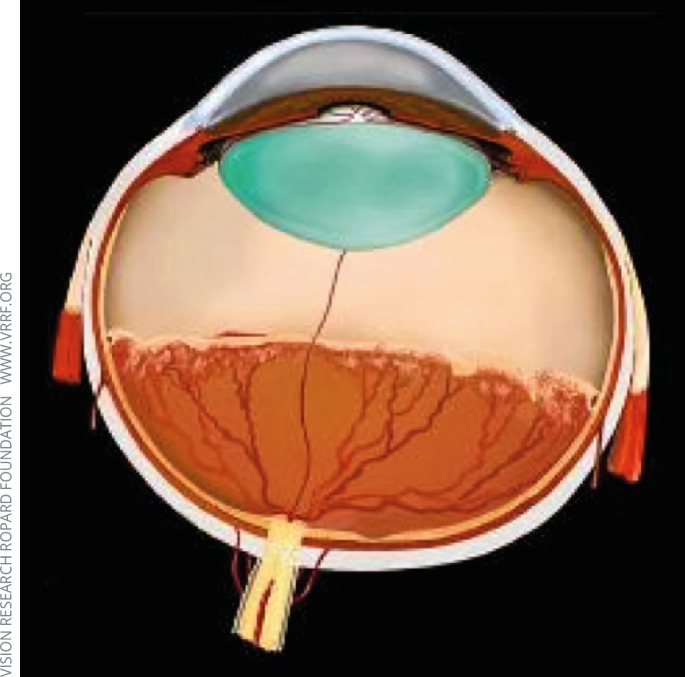
ROP occurs at the junction between the vascularised and unvascularised retina

In babies who are born preterm (before 37 weeks), the retinal blood vessels are not fully formed and do not reach the ora serrata (Figure 2). If a preterm baby is examined a week or so after birth, it is possible to see whether the blood vessels are mature and have reached the ora serrata, or whether they are immature; i.e., the peripheral retina is not vascularised. If babies receive good neonatal care, the retinal blood vessels continue to grow normally. If the neonatal environment is not ideal, particularly if oxygen levels have been higher or more variable than they should be, the retinal blood vessels stop growing. A visible line or a ridge then forms and the blood vessels may start to multiply (proliferate) abnormally. The visible line, ridge and proliferating blood vessels are all signs of retinopathy of prematurity (ROP). See Figure 3.

**Figure 4:**
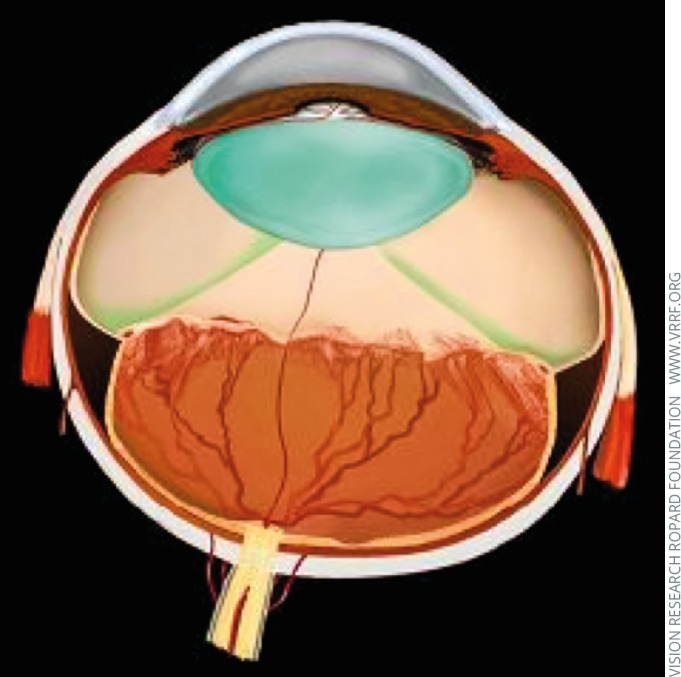
Advanced ROP with partial retinal detachment

In 5–10% of premature babies, ROP progresses and can lead to retinal detachment (Figure 4). This causes irreversible blindness, often in both eyes.

